# Automated workflow for characterization of bacteriocin production in natural producers *Lactococcus lactis* and *Latilactobacillus sakei*

**DOI:** 10.1186/s12934-024-02349-6

**Published:** 2024-03-03

**Authors:** Valentin Steier, Lisa Prigolovkin, Alexander Reiter, Tobias Neddermann, Wolfgang Wiechert, Sebastian J. Reich, Christian U. Riedel, Marco Oldiges

**Affiliations:** 1 Institute of Bio- and Geosciences, IBG-1: Biotechnology, Forschungszentrum Jülich GmbH, Jülich, Germany; 2 Institute of Biotechnology, RWTH Aachen University, Aachen, Germany; 3 NovaTaste Production GmbH, Holdorf, Germany; 4 Computational Systems Biotechnology (AVT.CSB), RWTH Aachen University, Aachen, Germany; 5 Department of Biology, Ulm University, Ulm, Germany

**Keywords:** Bacteriocins, *L. lactis*, *L. sakei*, Microcultivation, Laboratory automation

## Abstract

**Background:**

Lactic acid bacteria are commonly used as protective starter cultures in food products. Among their beneficial effects is the production of ribosomally synthesized peptides termed bacteriocins that kill or inhibit food-spoiling bacteria and pathogens, e.g., members of the *Listeria* species. As new bacteriocins and producer strains are being discovered rapidly, modern automated methods for strain evaluation and bioprocess development are required to accelerate screening and development processes.

**Results:**

In this study, we developed an automated workflow for screening and bioprocess optimization for bacteriocin producing lactic acid bacteria, consisting of microcultivation, sample processing and automated antimicrobial activity assay. We implemented sample processing workflows to minimize bacteriocin adsorption to producer cells via addition of Tween 80 and divalent cations to the cultivation media as well as acidification of culture broth prior to cell separation. Moreover, we demonstrated the applicability of the automated workflow to analyze influence of media components such as MES buffer or yeast extract for bacteriocin producers *Lactococcus lactis* B1629 and *Latilactobacillus sakei* A1608.

**Conclusions:**

Our automated workflow provides advanced possibilities to accelerate screening and bioprocess optimization for natural bacteriocin producers. Based on its modular concept, adaptations for other strains, bacteriocin products and applications are easily carried out and a unique tool to support bacteriocin research and bioprocess development is provided.

**Supplementary Information:**

The online version contains supplementary material available at 10.1186/s12934-024-02349-6.

## Background

The United Nations document “2030 Agenda for Sustainable Development” highlights 17 Sustainable Development Goals (SDGs) containing, among others, “SDG 2: Zero Hunger” and “SDG 3: Good Health and Well-Being” [1]. According to sources that include the World Health Organization (WHO), at least a tenth of the world’s population is estimated to be affected by foodborne illnesses, causing large amounts of food to be discarded [2]. One such foodborne illness is listeriosis, a systemic infection caused by pathogenic *Listeria* species that has severe and potentially lethal effects on human health [3]. A favored strategy for food preservation is the so-called hurdle technology, where several measures with ideally orthogonal mechanisms are put into place to act against food pathogens, aiming to avoid foodborne illnesses and food spoilage [4].

Bacteriocins are ribosomally synthesized peptides exhibiting antimicrobial activity against a wide range of Gram-positive bacteria [5]. They are natural products of some bacteria, e.g., *Lactococcus* and *Latilactobacillus* species and are used in food preservation, while simultaneously meeting the demand for natural, minimally processed foods. They have been shown to be especially useful as an additional hurdle in the case of *Listeria* [6], which can grow even at refrigerator temperatures [7]. Usually, these bacteria are added as protective starter cultures for food products. Their ability for fermentation in the food matrix leads to a decrease in pH value, metabolization of nutrients, formation of aroma components, and decrease in redox potential, thus improving the sensory quality of the given food product including flavor and texture [8]. Simultaneously, their natural production of bacteriocins and organic acids as well as the competition for nutrients, leading to competitive exclusion of other organisms, contribute to increase product shelf-life and consumer safety [9]. Many bacteriocin-producing starter cultures used by the food industries belong to the lactic acid bacteria (LAB).

Due to its long history of safe use, nisin produced by *Lactococcus lactis* is a prominent example of a food additive with GRAS (Generally Recognized as Safe) status approved by the Food and Drug Administration (FDA) [10]. In recent years, the discovery of novel bacteriocins and their natural producer strains has been accelerated due to the ongoing improvement of modern biological methods and the availability of comprehensive genome databases [11]. The mechanism of action often remains unknown for many bacteriocins, although it is known that the majority exerts their antimicrobial activity via pore formation in the bacterial cell membrane [12]. Due to the different membrane structure, Gram-negative organisms are often resistant to bacteriocins, but can be rendered susceptible following addition of compounds impairing the outer membrane, such as EDTA [13]. As bacteriocins interact specifically with a bacterial target or a group of bacteria, screening for novel bacteriocins and their production strains is a promising approach to identify and characterize improved starter culture candidates for targeted applications in a broad range of food products [14]. However, due to the number of potential strain candidates and the necessary development cycles of bioprocess optimization, the resulting number of experiments can quickly exceed the capacity of common laboratory scale bioreactors. Here, automation and microcultivation provide essential tools to accelerate screening approaches, increasing the experimental throughput while simultaneously providing a high level of experimental insight [15].

Bacteriocin producing strains and processes are usually compared based on their antimicrobial activity against a chosen indicator strain. In literature, such antimicrobial activities are often measured using standard agar-plate based inhibition assays such as agar drop test, well diffusion assay or disk diffusion assay [16]. However, they are laborious and could lack reproducibility and robustness with regards to quantification as diffusion effects are not accounted for. Moreover, they often lack standardization of the operator procedure especially for the sample processing [17]. In particular, adsorption of bacteriocins to producer cells has direct consequence on antimicrobial activity determination and can be influenced by sample processing strategies [18]. Optical assays are a promising alternative to quantitatively evaluate antimicrobial activity [19] and are especially suited for automation with liquid handling systems. In order to make optimum use of automation, the manual processing and sample handling should be minimized. This leads to a reduction in equipment idle times and human errors, as well as an increase in autonomy and available personnel capacities.

In this contribution, we present an automated workflow with minimal human interaction for the cultivation and bioprocess development of natural bacteriocin producers. The workflow utilizes microcultivation technology for increased experimental throughput, automated sampling and sample processing strategies to improve reproducibility between processes and minimize human error. This is accompanied by a quantitative fluorimetric assay for robust antimicrobial activity measurement. We demonstrate the usefulness of the automated workflow to assess key process phenotypes for bacteriocin producing LAB, i.e., growth behavior, influence of pH and media components, i.e., buffer or yeast extract in a short time. Moreover, the workflow allows automated handling of sample material in a manner that avoids bacteriocin adsorption to producer cells, yielding a more reproducible and robust quantification of antimicrobial activity as the key performance indicator.

## Results

### Bacteriocin units (BU) as metric for antimicrobial activity

The characterization of bacteriocin producers and bioprocess optimization requires a performance indicator, usually based on the antimicrobial activity in the cultivation supernatant. This was achieved via use of the pHluorin2 assay, measuring the change in fluorescence ratio of the fluorescence protein pHluorin2 after a change in intracellular pH value caused by bacteriocin-induced pore formation in the cell membrane [19]. Here, a series of twofold sample dilutions up to a dilution factor of 2^8^ is added to an indicator strain sensitive to the bacteriocin, leading to a change of pHluorin2 fluorescence based on the corresponding antimicrobial activity or bacteriocin effect. To quantitatively compare different samples, several approaches are applied in literature such as the determination of minimal inhibitory concentrations (MIC) in such a dilution series based on a particular threshold for the antimicrobial activity of a sample [20–22].

**Fig. 1:**
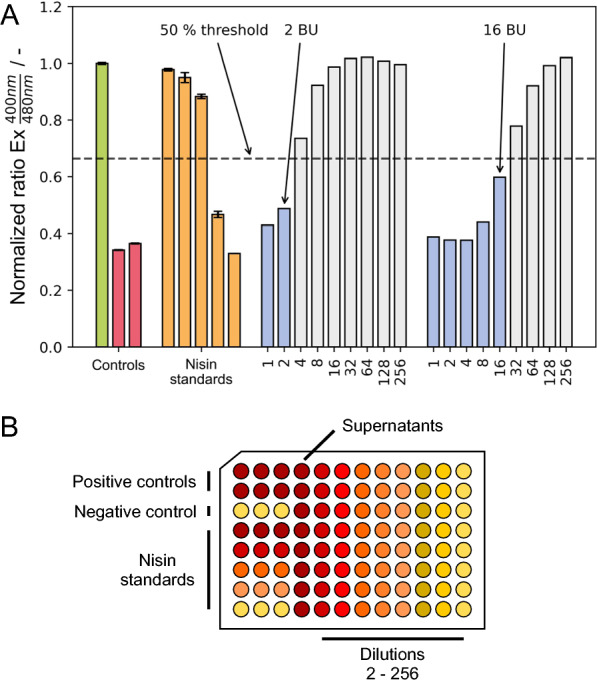
**A** Readouts of a pHluorin2 assay using sensor strain *L. innocua* LMG2785/pNZ-pHin2^*Lm*^ and determination of antimicrobial activity in bacteriocin units. Controls: Green—negative control (LMBO buffer); red—two positive controls (left: LMBO containing 0.01% CTAB, right: M17 complex medium diluted fourfold in LMBO containing 0.01% CTAB). Orange: LMBO containing nisin at 0.128, 0.5, 1.25, 2.5 or 5 µg mL^−1^ (from left to right). Blue/grey: exemplary cultivation supernatants obtained from *L. lactis* B1629. Blue: values below 50% threshold, grey: values above 50% threshold. 50% threshold is calculated from mean ratios of negative control and 5 µg mL^−1^ nisin. Controls and standards were measured in technical triplicates. Sample dilutions were measured in unicates. **B** Plate layout of assay MTP used in automated pHluorin2 assay.

Here, we adopted this approach to the pHlourin2 assay (Fig. 1A) and introduced the metric of the bacteriocin unit (BU), which is based on the fluorescence value of the positive (two red columns) and the negative control samples (green column) representing the upper and lower limit. Thus, it represents the theoretical 50% threshold between the signal of disrupted and live cell populations indicated by the dashed horizontal line taking into account the measured controls. Such a 50% threshold is often used in comparable methods described in literature (MIC, LD_50_). The two positive control samples were obtained from the used optimized listeria minimal buffer (LMBO) and M17 medium spiked with cetrimonium bromide (CTAB), respectively, both showing only very minor influence on the fluorescence readout. The necessary dilution factor for the individual sample leading to a fluorescence signal below the 50% threshold value represents the bacteriocin units, being a comparable metric between individual samples. With higher dilution factors possible, higher bacteriocin concentration can be deduced.

In the representative examples, obtained from cultivation with nisin Z producing strain *L. lactis* B1629, shown in Fig. 1 (blue and grey columns), the twofold dilution of the left sample dilution series lies below the threshold value indicated by the dashed line i.e., leading to BU = 2 for this sample. For the right dilution series of a different sample, the 16-fold diluted sample is below the threshold, leading to an antimicrobial activity of 16 BU. In analogy, the same approach can be used to evaluate the antimicrobial activity of sakacin A or any other pore-forming bacteriocin.

### Automation of pHluorin2 assay

**Fig. 2:**
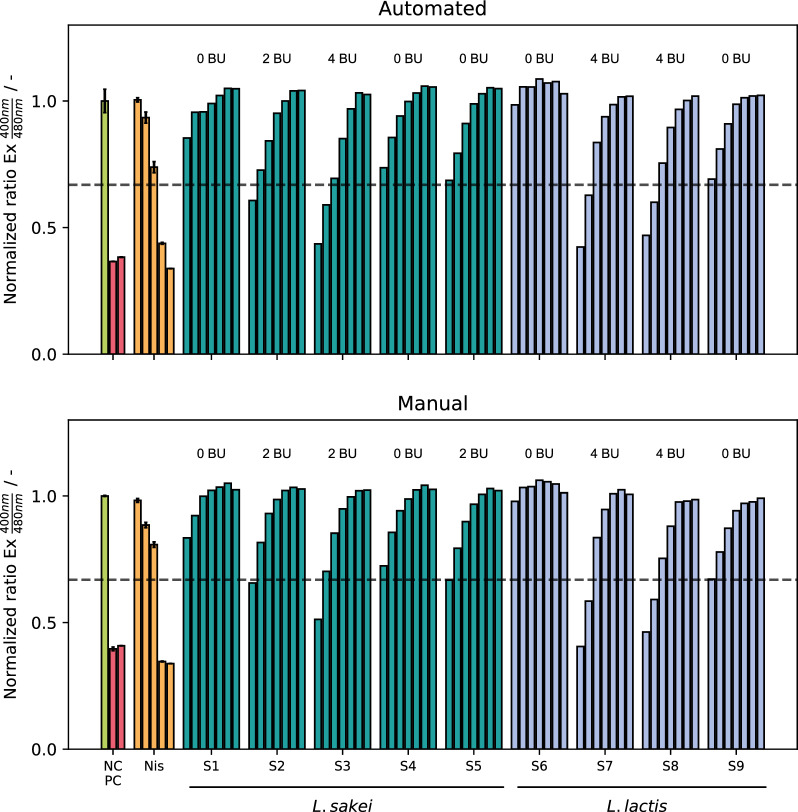
Comparison of automated (upper panel) and manual (lower panel) pHluorin2 assay results of nine random samples of supernatants of *L. sakei* A1608 (S1-S5) or *L. lactis* B1629 using sensor strain *L. innocua* LMG2785/pNZ-pHin2^*Lm*^ for determination of antimicrobial activity in bacteriocin units. NC/PC: Green—negative control (LMBO buffer); red—positive controls (left: LMBO containing 0.01% CTAB, right: M17 complex medium diluted fourfold in LMBO containing 0.01% CTAB). Nis: LMBO containing nisin in concentrations 0.128, 0.5, 1.25, 2.5 and 5 µg mL^−1^. Controls and standards were measured in technical triplicates. Sample dilutions were measured in unicates. 50% threshold is calculated from mean ratios of negative control and 5 µg mL^−1^ nisin. Measurements from undiluted cultivation supernatant were not analyzed due to interference of complex media with fluorescence measurement

Due to the large number of pipetting steps required to carry out the pHluorin2 assay including different controls, standards, and serial dilutions of samples, the assay is very laborious in terms of operator time. This holds especially true for larger sets of samples expected from parallelized microcultivation experiments, thus demanding automation of the assay. To establish a reliable assay readout, negative and positive controls as well as standards of commercial nisin in 5 different concentrations were measured in triplicates with every 96-well microtiter plate (MTP). Comparative evaluation of manual and automated pHluorin2 assay was done using cultivation samples from bioreactor cultivations of *L. lactis* B1629 and *L. sakei* A1608 (Fig. 2). Nine randomly selected samples were measured in twofold serial dilutions up to a dilution factor of 128 (2^7^) both in manual and automated fashion.

Very similar results could be obtained from manual and automated approach. In both cases, positive controls clearly indicate cell membrane disruption as they show a very low value, while negative controls show a high value, indicating live cell population. For 7 out of the 9 measured samples, the resulting antimicrobial activity in BU was identical. In two cases (samples S3, S5), the antimicrobial activity differed by only one dilution step, respectively. A closer look reveals that in fact only minor deviations in the calculated ratio led to the difference in resulting BU. This is pointing to an inherent drawback of every method using dilution series approaches relying on distinct threshold values, commonly used in many studies concerning antimicrobial or biological activity (e.g., MIC) [16]. If measurement values of samples are close to the threshold, a small signal variation can put the sample below or above the threshold value, showing demand for improvement in future calibration procedures. Overall, the automated and manual assay were in very good accordance.

**Fig. 3:**
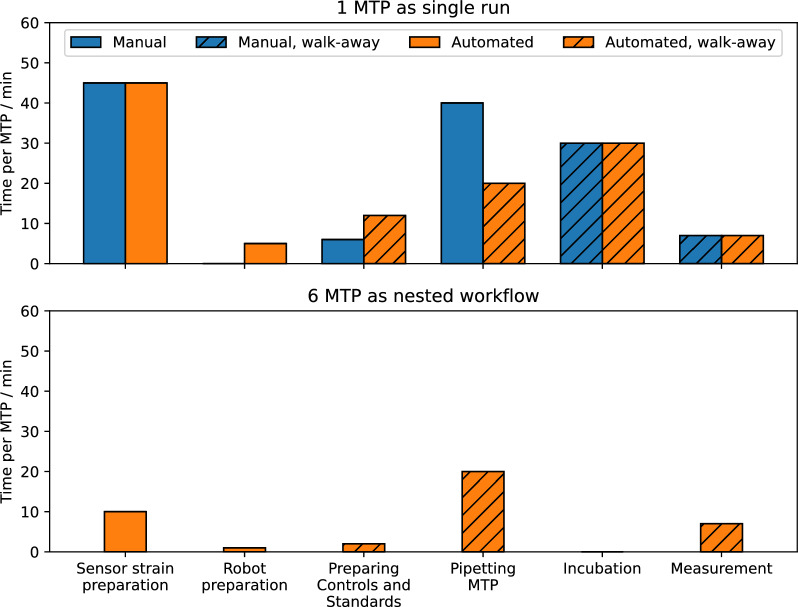
Overview of durations for automated and manual pHluorin2 assay showing the required time per MTP for the individual steps performed for one MTP as single run (top) and the optimized protocol for the nested workflow allowing to handle 48 samples from microcultivation (bottom). Sensor strain preparation: preparation of sensor strain cell suspension (see “Methods” section). Robot preparation: nesting refers to the performance of pipetting steps during incubation times. If suitable, manual pipetting steps were carried out using an 8-channel pipette

After successful setup of the automated pHluorin2 assay, further improvements were implemented to reduce manual operator time per sample and connect the workflow seamlessly with the microcultivation approach. The plate layout shown in Fig. 1B facilitates pipetting procedures for the liquid handling robotic arm to handle positive and negative controls, standards, and 8 samples in 9 dilutions within one 96-well MTP. The workflow was adapted to operate six 96-well MTP allowing parallel handling of all required controls, standards, and dilutions for 48 samples obtained from one microcultivation run. Furthermore, pipetting and incubating steps were nested to reduce the overall duration. Time savings can be achieved from the pipetting actions and incubation times (Fig. 3). While for one MTP, the total time decreases from 130 to 120 min only, the operator walk-away time increases from 40 to 70 min, i.e, increased from 30% to almost 60% of the total assay time. When measuring multiple MTP, the incubation steps can be nested with the pipetting steps, increasing the overall efficiency. The automated nested workflow for the measurement of six MTP substantially reduces the overall time per MTP from 120 to 40 min, while increasing the operator walk-away time up to 75%.

### Microcultivation workflow for bacteriocin producer strains

**Fig. 4:**
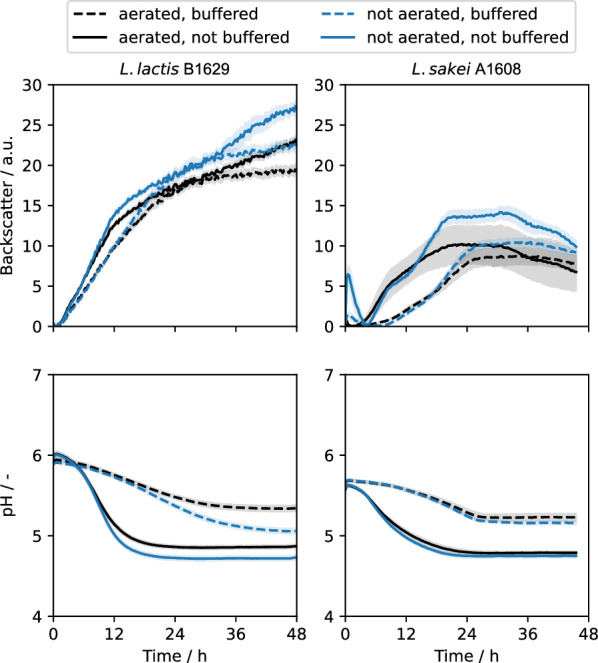
BioLector cultivations of *L. lactis* B1629 (left panels) and *L. sakei* A1608 (right panels) with measurements for biomass (backscatter in arbitrary units; a.u.; upper panels) and pH (lower panels). Cultivations were carried out in BOH3-RWP, non-aerated conditions were achieved by sealing plate with adhesive aluminum foil. M17 complex medium with 20 g L^−1^ glucose was used for *L. lactis* B1629, MRS medium was used for *L. sakei* A1608. MES buffer (pH 6.5) was added for buffering (0.2 M for *L. lactis* B1629, 0.1 M for *L. sakei* A1608). Cultivations were carried out at 30 °C, 600 rpm shaking frequency, 3 mm shaking diameter with humidity control (85%). Solid and dashed lines show the mean of 12 biological replicates with the blue and grey areas around representing standard deviation

The strains *L. lactis* B1629 [23], a natural producer of nisin Z, and *L. sakei* A1608, a natural producer of sakacin A, were used for the conception of the automated workflow since they are prominent representatives for natural bacteriocin producers belonging to the LAB. The presence of matured, dehydrated nisin Z [24] and matured sakacin A [25] in respective cultivation supernatants was confirmed via LC–MS measurement (Additional file 1: Figs. S1, S2). LAB are facultative anaerobes and cultivations with these strains are typically carried out with little to no agitation and often under microaerophilic to anaerobic cultivation conditions [26–28]. Here, growth can be inhibited by lactic acid production and decreased pH values [29]. Hence, the impact of cultivating these natural producers in the BioLector microcultivation device under aerated and non-aerated as well as buffered and non-buffered conditions were investigated (Fig. 4). Cultivations in the microcultivation device allow for online measurement of biomass and pH value in every well. Non-aerated conditions were achieved by sealing with adhesive aluminum foil, being non-permeable for gas. Since oxygen transfer can be neglected for non-aerated conditions, round well shaped microcultivation plates (RWP) and reduced shaking frequency of 600 rpm were used.

The repeatability between biological replicates (n = 12) in terms of time-course of backscatter signal and pH value was given for *L. lactis* B1629 (Fig. 4, left) and *L. sakei* A1608 (Fig. 4, right). Cultivation under non-aerated conditions yielded slightly higher final backscatter values. Cultivations carried out under buffered conditions with MES buffer showed a much more stable pH value throughout the course of the cultivation, but backscatter signals showed slightly slower increase, pointing to lower growth rates, which could be a consequence of higher salt content of the medium. For online pH measurement, the BOH3 type of the optode sensor spots was used providing a higher accuracy of the online pH measurement, specifically in the acidic range below pH 6. Standard BOH1-RWP used for neutral pH ranges were unable to clearly distinguish the buffered vs non-buffered conditions for both strains (data not shown).

### Automated sample processing workflow for bacteriocin producer strains

To integrate microcultivation into the developed automated workflow for antimicrobial activity determination via the pHluorin2 assay, automated sampling and sample processing strategies were required. The integration of the BioLector into the robotic platform allows for automated sampling throughout the cultivation based on several possible online triggers (time, backscatter, pH) with cell separation achieved automatically via a built-in centrifuge [30]. However, it was shown that bacteriocins could potentially adsorb to the cell envelope of the respective producer cells [18]. In such a case, measurement of antimicrobial activity in the cultivation supernatant would underestimate bacteriocin formation. Therefore, multiple approaches were tested to evaluate potential bacteriocin adsorption and ideally identify a strategy to ensure a reliable comparison of the measured antimicrobial activity of different samples. It was shown that the adsorption of bacteriocins to producer cells can be influenced by the addition of acid, base, divalent cations or surfactants [31–33]. As the investigation of these factors and their interactions result in a large number of possible combinations to test, the previously established protocols for LAB microcultivation and automated antimicrobial activity measurement were employed to enable a comprehensive study and to accelerate the progress. Cultivations were carried out using *L. lactis* B1629 in M17 complex media, supplemented with glucose (10 g L^−1^) and MES buffer (0.2 M, pH 6.5). Furthermore, cultivations were supplemented with Tween 80, CaCl_2_, and/or MgSO_4_ in different combinations to reduce potential bacteriocin adsorption during the cultivation with six replicates for every condition. Following cultivation, one sample of each condition was set to the pH value of 1, 2, 3, 4, 5, and 6, respectively, using 6 M HCl or 8 M NaOH and incubated for 30 min at room temperature to investigate the probable effect of pH shift on bacteriocin adsorption. The samples were centrifuged to obtain cell-free supernatant and the pH was titrated back to pH 6.2 (being standard condition for pHluorin2 assay) using 8 M NaOH, followed by antimicrobial activity measurement using automated pHluorin2 assay.

**Fig. 5:**
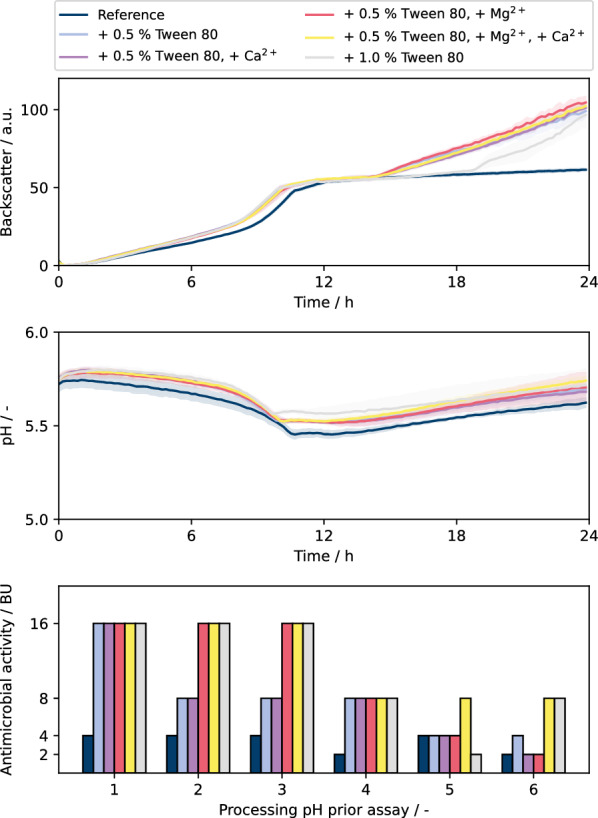
BioLector cultivations of *L. lactis* B1629. Cultivations were carried out in BOH3-RWP under non-aerated conditions. M17 complex media was used [10 g L^−1^ glucose, 0.2 M MES (pH 6.5)]. Cultivations were carried out at 30 °C, 600 rpm shaking frequency, 3 mm shaking diameter without humidity control. MgSO_4_ and CaCl_2_ were supplemented at final concentrations of 0.25 g L^−1^ and 0.5 g L^−1^, respectively. For backscatter and pH graph, solid lines show the mean of 6 biological replicates with the colored areas around representing standard deviation. Antimicrobial activities were determined in unicates using the pHluorin2 assay with sensor strain *L. innocua* LMG2785/pNZ-pHin2^*Lm*^. pH 1–6 indicates the pH value at which culture broth was incubated prior to cell separation. All supernatants were set to pH 6.2 prior to antimicrobial activity measurement

Again, the repeatability between biological replicates (n = 6) was very high regarding online measurements of backscatter and pH for the reference cultivation as well as for the different pH values with the three additives (Fig. 5). The replicates for the same condition show very good repeatability, and also the replicates of the different pH conditions were very similar. This demonstrates the excellent applicability of the microscale cultivation approach for LAB (Fig. 5). Except for the reference cultivation without any addition of Tween 80 or divalent cations, all other conditions seemed to show a slightly increased growth rate, but resulted in similar final biomass concentrations (Fig. 5, top). This is coherent with previously published results attributing a beneficial effect of Tween 80 on the growth of LAB up to a certain concentration [34, 35]. Strikingly, except for the reference cultivation all conditions seemed to show a second linear increase in backscatter value after 14 h. This can be attributed to the formation of visible aggregates in the presence of Tween 80 in the stationary phase, which obviously influenced the scattered light measurement. A possible reason for this could be partial cell lysis followed by aggregation of cell debris with molecules of Tween 80.

Regarding the antimicrobial activity, large differences were found ranging from BU = 2 up to 16, clearly demonstrating that adsorption of bacteriocins to producer cells seems to be an issue. The change from BU = 2 to 16 represents a substantial increase by a factor of 8. Interestingly, according to the reference data (Fig. 5, bottom), the temporary shift of pH during the sample processing seems to have very little influence with BU = 2–4. By contrast, conditions with Tween 80 and divalent cations added already during the cultivation showed much increased BU values of up to 16. A possible reason for the positive effect of Tween 80 may be increased hydrophobic interaction, reducing the adsorption of non-polar peptides to the non-polar cell envelope. Very strikingly, such positive effect was highly connected to the pH shift applied during sample processing. While there is only small positive effect of Tween 80 and divalent cations at pH 5 and 6, there was strong increase for pH values in the range of 1 to 3. A low pH value could show a positive effect on the solubility of nisin Z [36]. One can conclude that there seems to be a strong synergistic effect between the addition of Tween 80 during cultivation and the pH shift during sample processing. With respect to the role of the divalent cations Mg^2+^ and Ca^2+^, the data do not show a clear picture. Since Tween 80 and pH shift seem to provide the major effect, a potential small effect of those cations is difficult to deduce. Positive impact of cation addition for the production of nisin Z with *L. lactis* has been reported [33]. The influence of Tween 80 in the cultivation especially in combination with lower pH values during sample processing became apparent, which is coherent with previously published results on this matter [18]. Synergistic effects with positive impact on antimicrobial activity have also been reported elsewhere [37]. As the incubation at low pH values was carried out after the cultivation had been completed, an impact on the biological production of nisin Z could be ruled out. It seems likely, that the increased antimicrobial activities observed are due to decreased level of bacteriocin adsorption to the producer cell envelope, as published by Yang et al. [18], although residual adsorption of bacteriocin is still possible. In contrast, Tween 80, MgSO_4_, and CaCl_2_ were added at the beginning of the cultivation. As the growth was affected positively, an impact on the production of nisin Z cannot be ruled out. However, the positive effect of Tween 80 to reduce adsorption of bacteriocins produced was clearly more pronounced in combination with pH shift during sample processing, thus suggesting a reduced adsorption behavior, rather than an increase of product formation. Furthermore, detergents such as Tween 80 can also help increase solubility of peptides, as well as prevent their aggregation, adding further possible causes for the observed effects [38].

These findings support the hypothesis of adsorption or aggregation effects occurring during the nisin Z production process using *L. lactis* B1629, which could mask the bacteriocin formation when testing different bioprocess parameters or strains. Such effects can be counteracted by addition of Tween 80 during the cultivation and acidic pH shift during sample processing in order to reliably evaluate the process performance. As a consequence, adsorption effects were minimized within the devised automated process development workflow via the implementation of several strategies. The addition of media components, such as Tween 80 or divalent cations, is straightforward to achieve during media preparation, i.e., independent from any technical requirements. However, the precise incubation of biomass samples at a lower pH value adds complexity to the automated pHluorin2 assay and was integrated into the existing workflow steps by preparing 96-well deep well plates (DWP) with defined volumes of 4 M H_2_SO_4_ (up to 75 µL) for acidification and 8 M NaOH (up to 50 µL) for neutralization. This strategy allows an automated incubation at low pH to minimize adsorption effects for the subsequent pHluorin2 assay. The liquid handling system can draw samples from the microcultivation device during the cultivation process, transfer them to the DWP, where the biomass of the sample is added to the previously pipetted defined sulphuric acid volume. After an incubation step at pH 1, the cells are automatically separated via centrifugation and the supernatant can be transferred to a second DWP with NaOH to be neutralized to pH 6.2 prior to antimicrobial activity measurement. These methods were implemented successfully with a maximal deviation from target pH value of 0.7 units. The volume of NaOH was selected such that neutral to basic pH values of the supernatant are avoided, which could lead to loss of activity by precipitation or degradation of nisin Z [36].

### Application of automated workflow to evaluate impact of buffer concentration

The main product of LAB during growth on glucose is lactic acid, which is metabolically required to balance redox cofactors, but can lead to inhibition associated with pH decrease in pH non-controlled cultivations or effects of lactate accumulation in late growth phase [29, 39]. Addition of buffers to cultivation medium can help prolong the growth phase by stabilizing the pH value. Usually, bacteriocin production occurs during microbial growth, making the pH value an important parameter for bacteriocin production processes. MES is a widely used, non-toxic buffer component with a p*K*_a_ value of 6.15 at 20 °C, making it very useful for microbial cultivations in pH ranges close to 6.0 [40, 41]. The impact of the addition of different concentrations of MES buffer to the cultivation medium to enhance buffer capacity was investigated for growth of *L. lactis* B1629 and nisin Z formation. All samples were processed automatically, including the aforementioned integrated incubation at a processing pH value of 1 prior to cell separation.

**Fig. 6:**
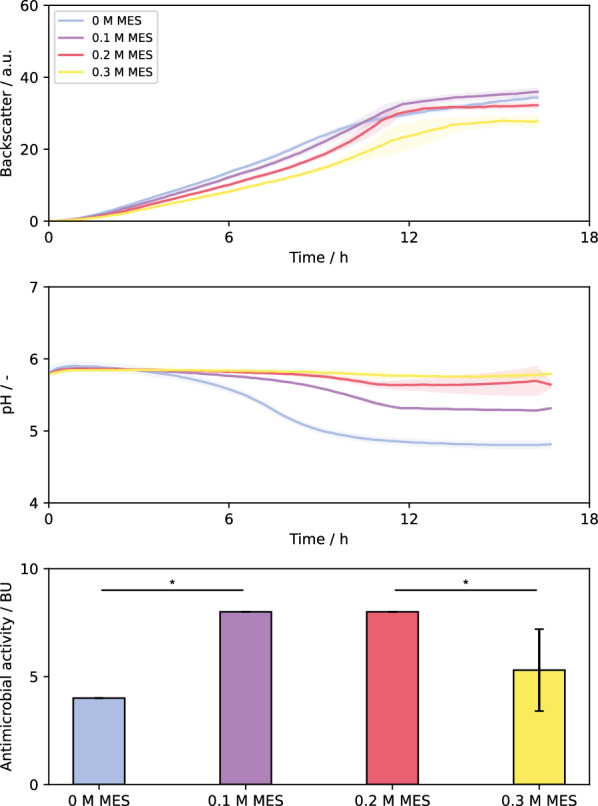
BioLector cultivations of *L. lactis* B1629. Cultivations were carried out in BOH3-RWP under non-aerated conditions. M17 complex media was used [10 g L^−1^ glucose, 0.5% (v/v) Tween 80]. Cultivations were carried out at 30 °C, 600 rpm shaking frequency, 3 mm shaking diameter without humidity control. Antimicrobial activities were determined using the pHluorin2 assay with sensor strain *L. innocua* LMG2785/pNZ-pHin2^*Lm*^. For backscatter and pH graph, solid lines show the mean of three biological replicates with the colored areas around representing standard deviation. Please note, that for MES concentration of 0, 0.1 and 0.2 the same BU values of 4 and 8 were measured for the three biological replicates, so that no standard deviation is visible. Statistical analysis was carried out via ANOVA with LSD post-hoc-test with α = 0.05. Significant differences are marked with *

At first, the impact of MES buffer concentration (0.1, 0.2 and 0.3 M) on growth, pH value and antimicrobial activity were investigated using *L. lactis* B1629 (Fig. 6). Not surprisingly, increasing concentration of MES buffer showed slower growth phenotype and lower final backscatter values which could be an effect of increased osmolality.

For cultures with 0.1 M MES or unbuffered cultures, the pH decreased throughout the course of the cultivation down to pH = 5.3 and 4.8, with a stronger decrease observed for unbuffered cultures. The addition of 0.2 M and 0.3 M MES led to similar pH profiles with final pH values of 5.6 and 5.8, i.e., 0.2 M MES seems to be the best compromise between growth phenotype and pH stabilization.

Besides pH stabilization, the addition of MES buffer increased antimicrobial activity with the highest activities observed for 0.1 and 0.2 M MES, demonstrating a positive impact on bacteriocin production and pH stabilization with only minor impact in terms of growth phenotype. The optima for growth of LAB and for bacteriocin production may differ according to literature, with lower pH values being beneficial for bacteriocin production [42, 43]. Of note, many pH studies do not distinguish between effects of pH on product formation or potential adsorption of readily formed product to bacteria. Moreover, pH control has been reported to be beneficial for bacteriocin production using LAB [27]. However, contradicting results depending on strains used have been reported [44].

### Transfer of automated workflow to *L. sakei* A1608

**Fig. 7:**
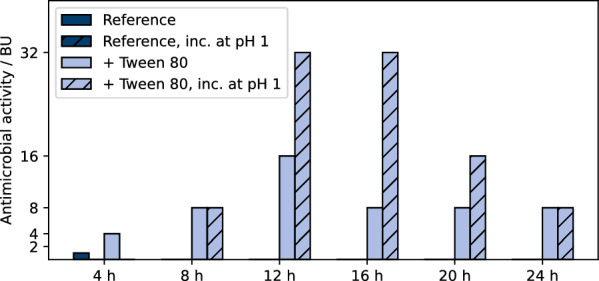
Antimicrobial activities determined in unicates via pHluorin2 assay using *L. innocua* LMG2785/pNZ-pHin2^*Lm*^ following BioLector cultivation of *L. sakei* A1608. Cultivations were carried out in BOH3-RWP under non-aerated conditions. MRS complex media was used. Cultivations were carried out at 30 °C, 600 rpm shaking frequency, 3 mm shaking diameter without humidity control. “inc. at pH 1” indicates incubation of biomass at a pH value of 1 prior to cell separation. "+ Tween 80" indicates supplementation of 0.5% (v/v) of Tween 80. The supernatant was set to pH 6.2 prior to antimicrobial activity measurement

The established automated process development workflow consisting of microcultivation, automated sampling and sample processing followed by antimicrobial activity measurement was then applied to sakacin A production using *L. sakei* A1608. As the extent of adsorption of bacteriocins to the producer cell might be specific to the given combination of bacteriocin and microorganism, the impact of sample processing required further investigation. As the addition of Tween 80 and the incubation of cell suspension samples at pH 1 had great impact on adsorption characteristics for *L. lactis* B1629, these strategies were also investigated for sakacin A production using *L. sakei* A1608. In this case, the effects were analyzed for multiple samples over the cultivation period of 24 h (Fig. 7).

In general, there was a clear difference in antimicrobial activity after addition of Tween 80 and pH shift to 1 similar to the experiments with *L. lactis* B1629. Highest antimicrobial activity showing 32 BU was found in samples after 12 and 16 h, followed by decline to 8 BU, most likely an effect of peptide aggregation or degradation entering the stationary phase (c.f. Fig. 7).

**Fig. 8:**
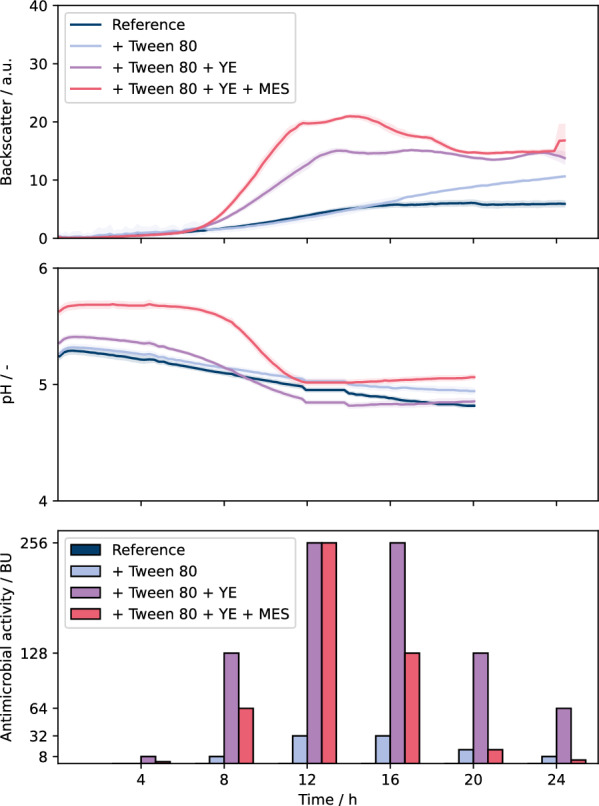
BioLector cultivations of *L. sakei* A1608. Cultivations were carried out in BOH3-RWP under non-aerated conditions. MRS complex media was used. Cultivations were carried out at 30 °C, 600 rpm shaking frequency, 3 mm shaking diameter without humidity control. Tween 80 was supplemented at final concentrations of 0.5% (v/v). YE: yeast extract was supplemented at a final concentrations of 20 g L^−1^. MES: MES buffer (pH 6.5) was supplemented at a final concentrations of 0.1 M. For backscatter and pH graph, solid lines show the mean of initially 12 biological replicates with the colored areas around representing standard deviation. The number of replicates decrease by two every 4 h of cultivation time due to sacrifice sampling. pH measurement experienced fallouts, missing values were extrapolated as mean of closest prior and posterior measurement value. Antimicrobial activities were determined in unicates using the pHluorin2 assay with sensor strain *L. innocua* LMG2785/pNZ-pHin2^*Lm*^

With the established automated workflow, the impact of the addition of MES buffer and yeast extract were analyzed. In general, a high repeatability of biological replicates was observed for all conditions, where addition of yeast extract and buffer led to significant positive effects on growth and peptide formation (Fig. 8). The addition of Tween 80 did not alter the growth behavior, but linear increase in backscatter signal appeared after growth phase at 16 h, which can again be attributed to the formation of light scattering aggregates associated with Tween 80. The addition of yeast extract led to substantially increased growth rate and final biomass concentration and both further increased with combined addition of yeast extract and MES buffer. The pH decreased throughout the course of the cultivation for all conditions. The stronger decrease of pH with increasing biomass is likely due to increasing amounts of lactic acid produced. Also, cultivations with 0.1 M MES showed pH decrease indicating that buffering capacity might still not be optimal. Regarding the antimicrobial activity, there is clear dependency on growth showing highest values of 256 BU right after exponential growth phase at 12 h, while the activity substantially dropped to 2–64 BU after 24 h [45, 46]. Strikingly, for the reference cultivation of *L. sakei* A1608 no antimicrobial activity and for cultivations with Tween 80 only small values up to 32 BU were measured after growth phase. One explanation for the low BU data is the low biomass concentration obtained from both conditions, pointing to some limitation of one or more media components in the complex MRS medium used. Yeast extract has been reported to be beneficial in bacteriocin production processes using LAB [47, 48]. The addition of 20 g L^−1^ yeast extract boosted both growth as well as formation of antimicrobial activity up to 256 BU after 12 h. Further addition of MES buffer led to higher biomass concentration, but antimicrobial activity was not increased. Instead, it seems that the buffered cultivation lost antimicrobial activity in the stationary phase faster. This could be some indication about sakacin A instability due to proteolytic degradation at higher pH values, which has also been observed during bacteriocin production processes [26].

These results reveal that the measured antimicrobial activity is not just dependent on the growth phenotype, as the buffered conditions reached the highest biomass concentrations, but the antimicrobial activities were not higher. Here, the pH value may have also influenced the bacteriocin production, as it was higher compared to those without buffer, which appears to have improved growth, but not necessarily bacteriocin production.

## Conclusion

The value of bacteriocins and their applications has risen in recent years and has led to increasing efforts to find novel bacteriocins and improve their production processes. Here, we present an automated workflow that enables accelerated strain screening and process development for bacteriocin production with LAB. The workflow consists of microcultivation in the BioLector device, followed by automated sampling and sample processing to minimize adsorption effects and subsequent antimicrobial activity measurement via the automated pHluorin2 assay. The whole workflow is set up to facilitate 48 replicates and can run with almost complete autonomy, except for the preparation of the BioLector cultivation plate and the provision of the sensor strain cell suspension for the pHluorin2 assay. Growth and bacteriocin production phenotypes have been analyzed for *L. lactis* B1629, a natural producer of nisin Z and *L. sakei* A1608, a natural producer of sakacin A. After successful development of microcultivation protocols and automation of the pHluorin2 assay, a suitable sample processing method was implemented to evaluate and minimize bacteriocin cell adsorption. By supplementing Tween 80 and setting an acidic environment, synergistic effects of both conditions were observed. These implementations facilitated quantitative comparison between different bioprocess parameters, such as between buffered and non-buffered cultivation without interference through their impact on adsorption behavior. Generally, the workflow is very versatile and can be tailored to varying strains, processes and degree of insight. This was demonstrated by different sampling strategies such as end-point sampling for up to 48 replicates and time-resolved sampling throughout a cultivation period. The latter was used for monitoring sakacin A production with *L. sakei* A1608, confirming growth-associated increase of antimicrobial activity followed by a most likely proteolytic degradation after the onset of the stationary phase. This allows the users to carry out both strain screening of larger strain libraries as well as a subsequent bioprocess optimization with a subset of strains.

The workflow represents a basis for further research on bacteriocin production processes. Due to the physicochemical variability of different bacteriocins, pH titration or peptide stability at given pH values need to be considered in any workflow to minimize peptide degradation. Although the workflow utilizes automation to a high degree, the manual process set-up and strain as well as pre-culture management remain challenges for future work.

In general, the workflow is designed to be a modular solution to accommodate potential other changes depending on the application. It allows easy adaptation with respect to cultivation of different strains and production of various bacteriocins. Of note, the pHluorin2 assay only addresses membrane-targeting bacteriocins due to its inherent properties of the sensor strains. However, sensor bacteria can be selected from readily available strains [19, 49] or easily generated de novo depending on the produced bacteriocin. Also, other assays evaluating the antimicrobial activity could be incorporated into the workflow, as long as they are compatible with standard labware.

## Methods

### Bacteriocin producing strains

Nisin Z producing strain *Lactococcus lactis* ssp. *lactis* B1629 [23] and sakacin A producing strain *Latilactobacillus sakei* A1608 were used as natural bacteriocin producers. *L. sakei* A1608 was obtained from the strain collection of NovaTaste Production GmbH.

### Cultivation conditions

Cultivations of *L. lactis* B1629 were carried out in M17 complex medium (Merck, Germany), supplemented with varying amounts of different media components, if necessary. Nisin production was induced by supplementing nisin (2 ng mL^−1^) to the cultivation media. Cultivations of *L. sakei* A1608 were carried out in MRS complex medium (Carl Roth, Germany). For cryo-conservation, both strains were grown from a single colony and incubated statically at 30 °C, resuspended in NaCl solution (0.9%, w/v) with 25% glycerol at an optical density of 10 at 600 nm and stored at − 80 °C. Pre-cultures for both strains were carried out in 50 mL centrifugation tubes with 30 mL complex media (supplemented with 0.5% glucose for *L. lactis* B1629) inoculated to an optical density of 0.1 and incubated statically at 30 °C for 16 h. Prior to main culture inoculation, the pre-culture cells were centrifuged (10 min, 4000×*g*, 4 °C) and resuspended in 0.9% (w/v) NaCl solution. Main cultures were inoculated to an optical density of 0.1. To prevent dilution through supplementation, a 2× stock of complex media was used for BioLector cultivations. BioLector cultivations were carried out with a well volume of 1 mL in BioLector Pro device (Beckman Coulter, USA) in MTP of type MTP-R48-BOH 3 (Beckman Coulter, USA) sealed with adhesive aluminum foil (Greiner Bio-One, Germany) being non-permeable for gas, if not mentioned otherwise. BioLector cultivations were carried out at 30 °C, 600 rpm shaking frequency and a shaking diameter of 3 mm without humidity control, if not mentioned otherwise.

### Biomass and cultivation measurements

Biomass measurements during shake flask or bioreactor cultivations was carried out using optical density measurement at 600 nm. During BioLector cultivations, integrated online scattered light measurement was used. Manually taken samples were centrifuged at 21,500×*g* at 4 °C for 10 min.

### Mass spectrometry

UPLC/MS-grade 0.1% formic acid in H_2_O and acetonitrile were obtained from Biosolve BV (Valkenswaard, Netherlands). LC–MS-grade H_2_O was obtained from a Milli-Q water purification system (Merck Millipore, Burlington, MA, USA). Proteome analysis was performed based on the method described in [50]. Supernatants were diluted 1:10 with LC–MS-grade H_2_O prior LC–MS measurements.

Liquid chromatography mass spectrometry (LC–MS) was conducted with an Agilent 1260 Infinity system (Agilent Technologies, Germany) coupled to a quadrupole time-of-flight mass spectrometer (TripleTOF6600, AB Sciex, Germany). LC was performed with an Ascentis® Express Peptide ES-C18, 2.7 μm HPLC column (53307-U, Merck, Germany) with a flow rate of 200 µL min^−1^ and the mobile phases (A) 0.1% formic acid in water and (B) acetonitrile. The elution gradient was as follows: 0 min, 3% B; 70 min, 40% B; 78 min 40% B, 79 min 60% B, 89 min 60% B, 90 min 3% B followed by a 12 min equilibration time between injections. Column temperature was set to 21 °C and injection volume to 20 µL. MS was conducted with a TurboV ion source operated in positive ionization mode. Ion spray voltage was set to 5.5 kV, source temperature to 450 °C, curtain gas to 35 psi, and the support gases GS1/GS2 to 50 psi/50 psi. All gases were nitrogen.

For intact peptide analysis, the quadrupole time-of-flight (QToF) mass spectrometer was operated in ToF mode with a dwell time of 250 ms and mass tolerance to 25 ppm. Acquired mass spectra were analyzed with PeakView 2.1 (AB Sciex, Germany).

### pHluorin2 antimicrobial activity assay

The pHluorin2 assay was carried out largely as previously published using *Listeria innocua* LMG2785/pNZ-pHin2^*Lm*^ [49] as biosensor. LMBO was used containing 200 mM MES, 4.82 mM KH_2_PO_4_, 11.55 mM Na_2_HPO_4_, 1.7 mM MgSO_4_, 4.54 mM (NH_4_)_2_SO_4_, 55 mM glucose, and set to a pH value of 6.2. All measurements were carried out using 100 µL of sensor strain cell suspension with an optical density set to 3.0 and 100 µL sample. CTAB was used as positive control, LMBO was used as negative control and commercial nisin was used as standard in concentrations of 0.128, 0.5, 1.25, 2.5 and 5 µg mL^−1^. Emission at 520 nm was measured at excitation wavelengths of 400 and 480 nm. To ensure comparability between different plate readers, any calculated emission ratio between 400 and 480 nm excitation on a given MTP was divided by the mean emission ratio calculated for the three negative controls (LMBO buffer). Antimicrobial activity measurements of cultivation supernatants were carried out in serial twofold dilutions up to a dilution factor of 256 (2^8^). The calculations were carried out using Python 3.10 with the packages numpy, pandas, and scipy.

### Robotic workflows and data analysis

All robotic workflows were carried out on a Freedom Evo Liquid Handler (Tecan, Switzerland) with integrated centrifuge (Hettich, Germany) and plate reader (Tecan, Switzerland). Experiments, which combined microcultivation and robotic workflows, were conducted using an in-house device control system. This system was used to continuously monitor BioLector data using the Python package bletl [51] and carry out sampling and sample processing workflows based on previously defined triggers (e.g., cultivation time). The necessary pipetting worklists were written with Python package robotools [52]. During automated sampling procedures, 800 µL sample were taken from the given cultivation well, added to a DWP that contained a defined amount (up to 75 µL) of 4 M sulphuric acid and incubated for 30 min, if necessary. After incubation, the samples were centrifuged at 4000×*g* at 4 °C for 6 min. 500 µL supernatant was transferred to a DWP containing a defined amount (up to 50 µL) of 8 M NaOH, if necessary. The supernatants were placed on a cooled tray on the robotic deck and analyzed for antimicrobial activity.

### Supplementary Information

**Additional file 1: Figure S1.** LC–MS analysis of cultivation supernatant of *L. lactis* B1629. **Figure S2.** LC–MS analysis of cultivation supernatant of *L. sakei* A1608.

## Data Availability

All data generated or analyzed during this study are included in this published article and its Additional file.

## Abbreviations

BU: Bacteriocin unit
CTAB: Cetrimonium bromide
DWP: Deep well plate
FDA: Food and Drug Administration
GRAS: Generally Recognized as Safe
LAB: Lactic acid bacteria
LMBO: Optimized listeria minimal buffer
MES: 2-(*N*-Morpholino)ethanesulfonic acid
MIC: Minimal inhibitory concentration
MTP: Microtiter plate
QToF: Quadrupole time-of-flight
RWP: Round well plate
SDGs: Sustainable development goals
WHO: World Health Organization
