# Supplementary material for: Automated workflow for characterization of bacteriocin production in natural producers Lactococcus lactis and Latilactobacillus sakei
Source: Microb Cell Fact. 2024 Mar 3;23:74. doi: 10.1186/s12934-024-02349-6 (PMC10910668; doi:10.1186/s12934-024-02349-6)
Supplement: Supplementary file 1 — Additional file 1: Figure S1. LC–MS analysis of cultivation supernatant of L. lactis B1629. Figure S2. LC–MS analysis of cultivation supernatant of L. sakei A1608. [file 12934_2024_2349_MOESM1_ESM.pdf]

Supplementary information to article "Automated workflow for characterization of bacteriocin production in natural producers *Lactococcus lactis* and *Latilactobacillus sakei*" (Steier et al.)

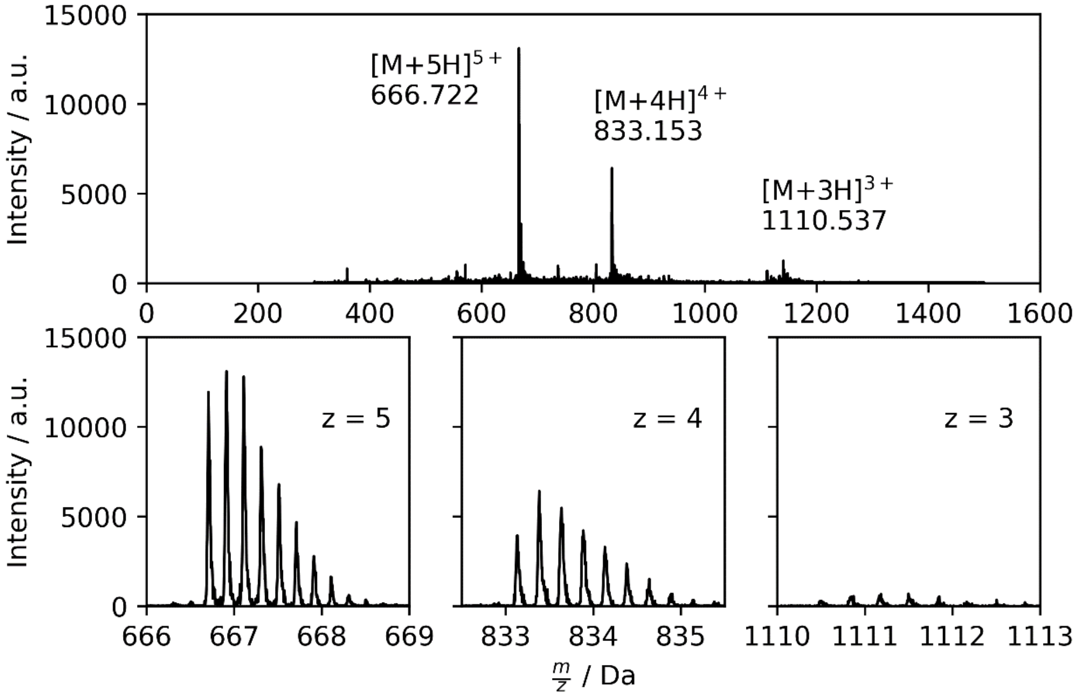

Figure S1: LC-MS analysis of cultivation supernatant of *L. lactis* B1629. The peaks with mass to charge ratios (m/z) of 1110.537, 833.153 and 666.722 correspond to mature nisin Z (no leader peptide; ITSISLCTPGCKTGALMGCNMKTATCNCSIHVSK; [M] = 3472.61 Da) with eight dehydrations on serine and threonine ([M] = 3328.49 Da) with three, four or five positive charges (top). Peaks displayed in the bottom figures correspond to enlarged representation of the three peptide species, showing natural isotope distribution.

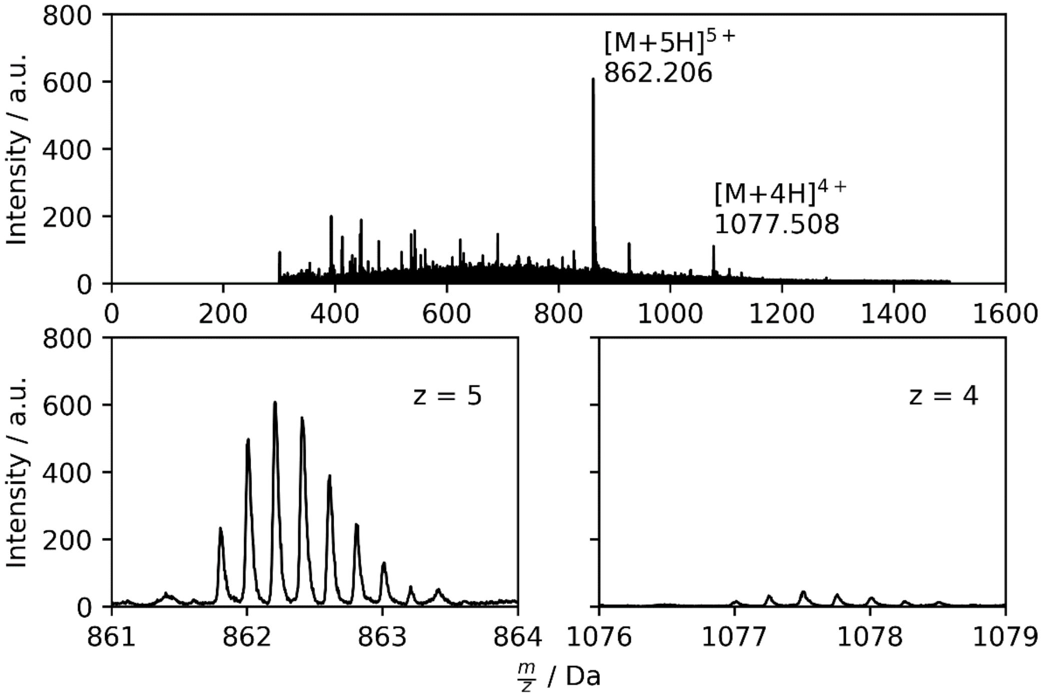

Figure S2: LC-MS analysis of cultivation supernatant of *L. sakei* A1608. The peaks with mass to charge ratios (m/z) of 1077.508 and 862.206 correspond to mature sakacin A (no leader peptide; ARSYGNGVYCNNKKCWVNRGEATQSIIGGMISGWASGLAGM; [M] = 4306.03 Da) with four or five positive charges (top). Peaks displayed in the bottom figures correspond to enlarged representation of the three peptide species, showing natural isotope distribution.
